# Early warning signals of Atlantic Meridional Overturning Circulation collapse in a fully coupled climate model

**DOI:** 10.1038/ncomms6752

**Published:** 2014-12-08

**Authors:** Chris A. Boulton, Lesley C. Allison, Timothy M. Lenton

**Affiliations:** 1 Earth System Science, College of Life and Environmental Sciences, Laver Building (Level 7), University of Exeter, Exeter EX4 4QE, UK

## Abstract

The Atlantic Meridional Overturning Circulation (AMOC) exhibits two stable states in models of varying complexity. Shifts between alternative AMOC states are thought to have played a role in past abrupt climate changes, but the proximity of the climate system to a threshold for future AMOC collapse is unknown. Generic early warning signals of critical slowing down before AMOC collapse have been found in climate models of low and intermediate complexity. Here we show that early warning signals of AMOC collapse are present in a fully coupled atmosphere-ocean general circulation model, subject to a freshwater hosing experiment. The statistical significance of signals of increasing lag-1 autocorrelation and variance vary with latitude. They give up to 250 years warning before AMOC collapse, after ~550 years of monitoring. Future work is needed to clarify suggested dynamical mechanisms driving critical slowing down as the AMOC collapse is approached.

## Abstract (web summary)

The ability to predict a potential collapse of the Atlantic Meridional Overturning Circulation is widely desired, but problematic. Here, using a fully coupled atmosphere-ocean general circulation mode, the authors show that early warning signs can be detected 250 years in advance.

## Graphical abstract

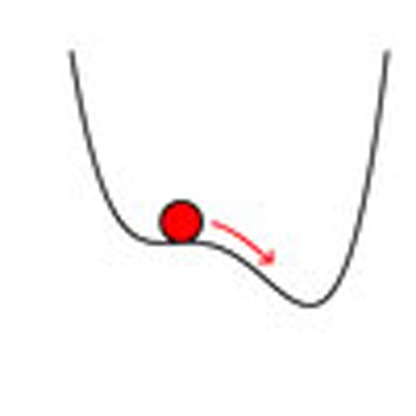

The Atlantic Meridional Overturning Circulation (AMOC) is a key component of the global climate system, responsible for a large fraction of the 1.3 PW northward heat transport in the Atlantic basin^1^. Numerical modelling experiments suggest that without a vigorous AMOC, surface air temperature in the North Atlantic region would cool by around 1–3 °C, with enhanced local cooling of up to 8 °C in regions with large sea-ice changes^2^,^3^. Substantial weakening of the AMOC would also cause a southward shift of the inter-tropical convergence zone, encouraging Sahelian drought^2^,^4^, and dynamic changes in sea level of up to 80 cm along the coasts of North America and Europe^5^,^6^. Theoretical arguments^7^, numerical models of varying complexity^8^,^9^,^10^,^11^ and evidence from palaeoclimate proxy records^12^,^13^ support the existence of two stable AMOC states—‘on’ and ‘off’. A reduction in density of the surface waters of the North Atlantic (through an increase in freshwater input or surface warming) can inhibit the formation of deep water and weaken the AMOC. In some model states, a weakening of the AMOC can result in an increase of freshwater transport into the Atlantic, resulting in a positive feedback. Numerical model projections suggest that the AMOC is likely to weaken over the 21st century^14^, but the likelihood of an abrupt collapse is very uncertain^15^,^16^, partly because most state-of-the-art climate models used for future projections cannot yet correctly simulate past abrupt climate changes^17^,^18^. This has generated interest in the possibility that generic early warning signals could exist before abrupt AMOC transitions^9^,^19^,^20^,^21^, which might be diagnosed directly from data. However, existing studies of this issue have been restricted to models of low^19^ or intermediate^9^,^20^,^21^ complexity.

For a low-order dynamical system approaching a threshold where its current state becomes unstable and it transitions to some other state, one can expect to see it become more sluggish in its response to small perturbations^20^. This phenomenon of ‘critical slowing down’ (CSD) can be detected in time series as increasing autocorrelation over time, measured by estimating the AR(1) coefficient (see Methods). Variance is also expected to increase^22^ (noting that this is not independent of lag-1 autocorrelation). Here we analyse data from simulations of the fully coupled climate model FAMOUS^23^. This is a lower resolution version of HadCM3, one of the models used in the IPCC Fourth Assessment Report. We look for early warning indicators in both the annual and decadal mean time series at 33.75°S–58.75°N (at every 2.5°). The time series of AMOC strength are cutoff at 800 years, before AMOC collapse, and are detrended before calculating the CSD indicators (see Methods). Throughout our analysis, Kendall’s τ rank correlation coefficient is used as a measure of tendency of the indicators. A value of τ=1 implies an indicator is always rising, τ=−1, always decreasing and τ=0 having no trend (see Methods).

## Results

### The AMOC in FAMOUS

**Figure 1:**
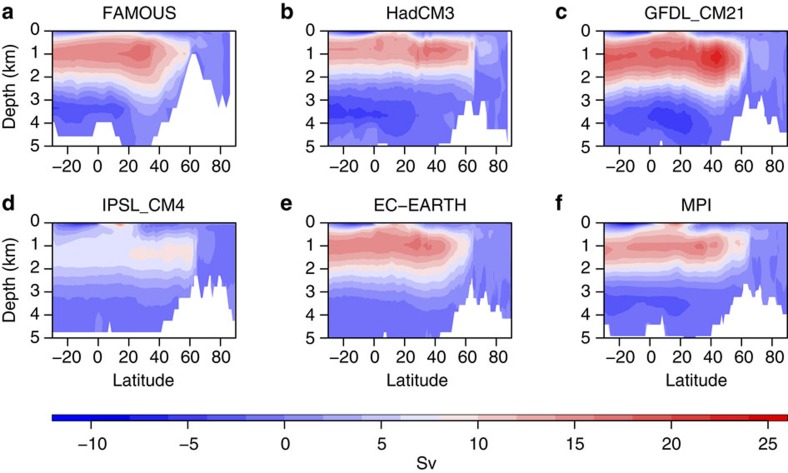
Mean AMOC streamfunction in FAMOUS and five other coupled AOGCMs. The streamfunction fields obtained from multicentennial control simulations are shown for (**a**) FAMOUS, (**b**) HadCM3, (**c**) GFDL_CM21, (**d**) IPSL_CM4, (**e**) EC_EARTH and (**f**) MPI.

**Figure 2:**
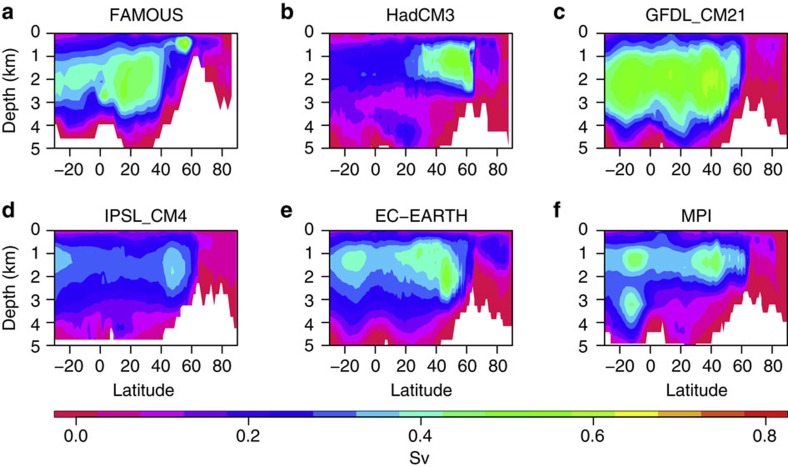
Local standard deviation in AMOC streamfunction on time scales longer than 50 years. The standard deviation in streamfunction is shown for (**a**) FAMOUS, (**b**) HadCM3, (**c**) GFDL_CM21, (**d**) IPSL_CM4, (**e**) EC_EARTH and (**f**) MPI.

The representation of the AMOC in FAMOUS is comparable to a variety of other fully coupled climate models (Figs 1, 2), all of which are of a higher spatial resolution, and several of which feature in the CMIP3 and CMIP5 data sets used in the IPCC assessments. The AMOC in FAMOUS is broadly similar to that in HadCM3 in terms of mean strength and decadal variability^23^. Higher frequency and interannual variability in FAMOUS behaves similarly to other models when compared with observations^24^. For variability on time scales longer than interannual, a comparison with observations is difficult due to the short observational record (currently around 10 years). Figure 1 shows the mean AMOC streamfunction averaged over multi-centennial control simulations of FAMOUS and five other coupled AOGCMs. Figure 2 shows that, although there is some inter-model variation, the magnitude of AMOC variability in FAMOUS on multi-decadal timescales is in the range of other models, both in terms of magnitude and pattern of variance. Furthermore, the mean transport at 26°N in FAMOUS compares well with the mean transport estimated from the RAPID/MOCHA/WBTS array^25^ over the years 2004–2012, where the AMOC is currently being monitored (both are ~17.5 Sv)^26^.

**Figure 3:**
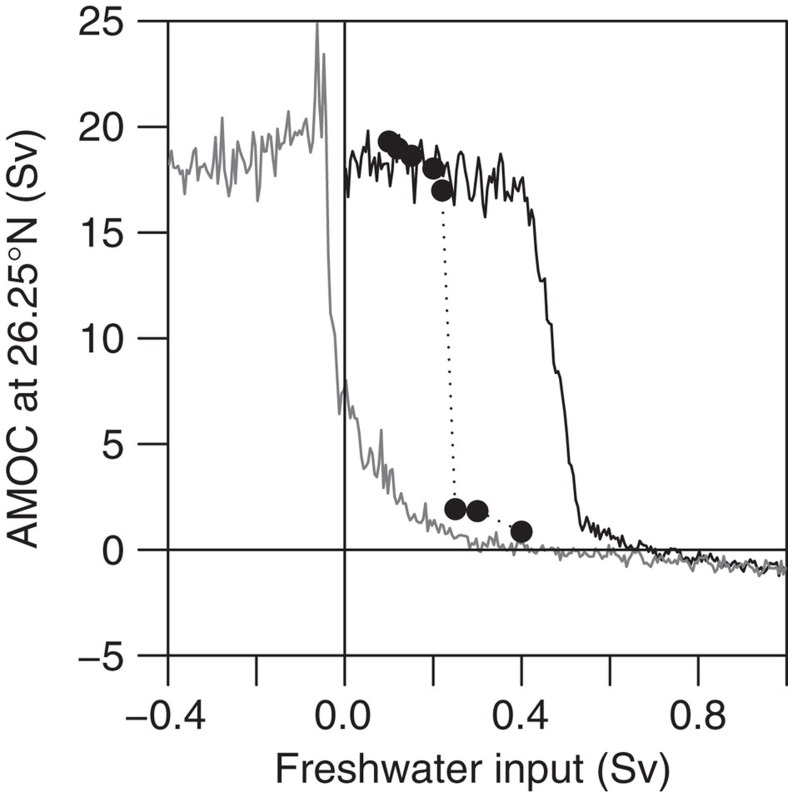
Hysteresis of AMOC in the FAMOUS model. AMOC transport (Sv) at 26.25°N and ~1,000 m depth is plotted as a function of imposed freshwater input. The solid black line shows the decadal mean AMOC during a transient experiment with freshwater input increasing from 0–1 Sv over 2,000 years. The solid grey line shows the same for freshwater input decreasing from 1 to −0.4 Sv at the same rate. The filled circles show the equilibrium AMOC transport reached during a series of constant-forcing simulations that are initialized from the corresponding point of the transient (increasing forcing) simulation. Adapted from Fig. 2 of ref. 11.

FAMOUS has been subjected to a hosing experiment^11^, where freshwater forcing is applied in the North Atlantic between 20°N and 50°N (and compensated with a spatially uniform salt flux to conserve global mean salinity). This acts to reduce the density of the surface waters, inhibiting the formation of North Atlantic Deep Water, and weakens the AMOC. This forcing is gradually increased at a rate of 5 × 10^−4^ Sv yr^−1^, eventually causing the AMOC to transition into the ‘off’ state after about 800 years of simulation, at a freshwater input of 0.4 Sv. The freshwater forcing is then removed at the same rate, allowing the AMOC to recover, with associated hysteresis (Fig. 3). Data from the simulation is saved at annual resolution, and we analyse just the upper branch of forcing towards AMOC collapse. We also make use of equilibrium simulations in our analysis (Fig. 3, black points and Results). These are initialized at particular values of forcing before the collapse occurs and run to equilibrium, either remaining in the ‘on’ state or eventually transitioning to the ‘off’ state.

### Early warning signals of AMOC collapse at annual resolution

**Figure 4:**
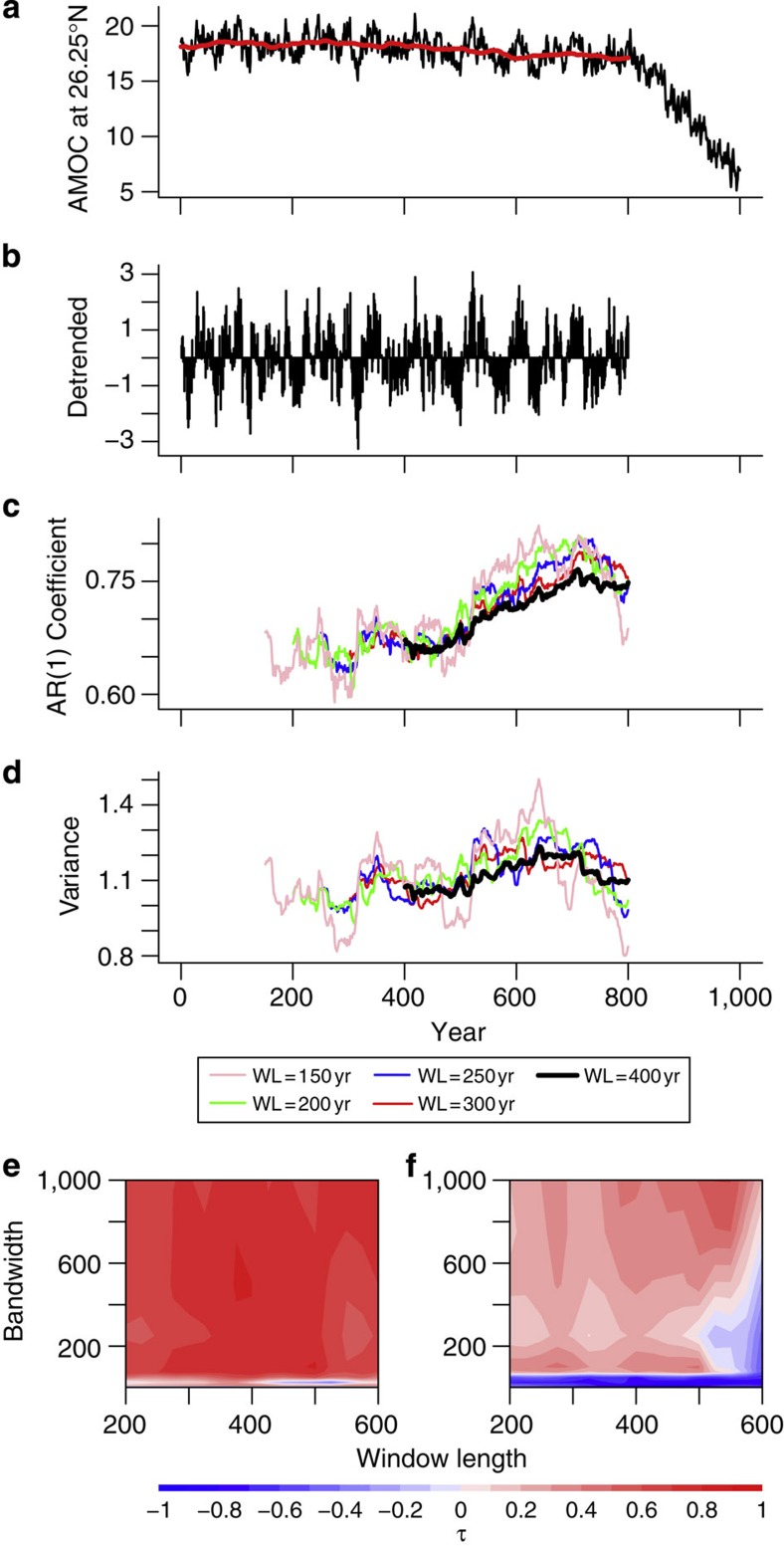
Early warning signals of AMOC collapse in the transient hosing experiment. Time series shown is from 26.25°N and ~1,000 m depth. (**a**) Annual time series up to red vertical line is detrended by a Kernal smoothing function (shown by smooth red line). The early warning signal analysis is carried out on (**b**) the residuals from this. Indicators of (**c**) AR(1) coefficient estimation and (**d**) variance are calculated as described in main text and Methods and plotted at the end of the window used to estimate them. Examples in (**c**) and (**d**) are shown for window lengths of 400, 300, 250, 200 and 150 years while using a detrending bandwidth of 100 years. Sensitivity analysis to determine how robust the indicators are to varying window length and detrending bandwidth is shown as contour plots of tendency, measured by Kendall’s τ (see Methods), in the window length-bandwidth plane for (**e**) AR(1) coefficient estimation and (**f**) variance.

We began our search for early warning signals of AMOC collapse at 26.25°N (Fig. 4a), near where an estimated reconstruction of the AMOC is currently monitored by the RAPID-WATCH/MOCHA/WBTS array in the real ocean^25^. Slowing down can be seen by eye in the detrended fluctuations of AMOC strength at this latitude (Fig. 4b). With a sliding window length of 400 years (black lines in Fig. 4c,d), AR(1) is found to be rising (with τ=0.79), with a less strong rise in variance (τ=0.39). These increasing trends in the indicators are generally robust to varying window length and detrending bandwidth (Fig. 4c–f). A decrease in variance seen when detrending using a low bandwidths (Fig. 4f) indicates a shift in power from high to lower frequencies that is consistent with CSD.

**Figure 5:**
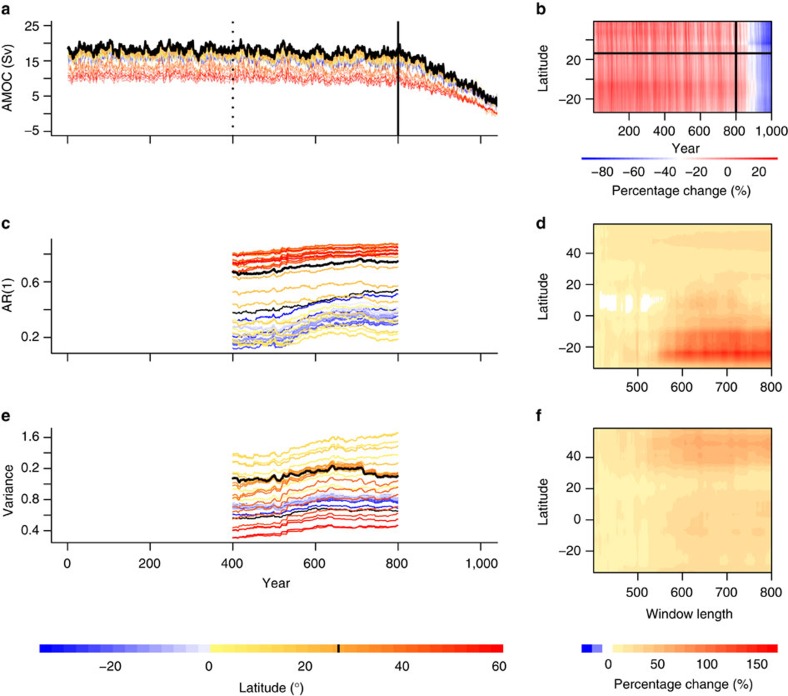
AMOC collapse and candidate early warning signals at each latitude. (**a**) Annual time series of AMOC (Sv) at each latitude are cut before collapse begins at 800 years (solid vertical line). (**b**) Time series are also shown as a contour plot in the time-latitude plane. They are then detrended and the analysis carried out on the residuals (see Methods, example in Fig. 4). A sliding window length of 400 years is used (dotted vertical line in (**a**) marks the end of the first window) to estimate candidate early warning signals (see Methods): (**c**,**d**) AR(1) coefficient, and (**e**,**f**) variance. Time series are coloured according to their latitude and 26.25°N (as in Fig. 4) is shown in black and contour plots of the percentage change are also shown.

**Figure 6:**
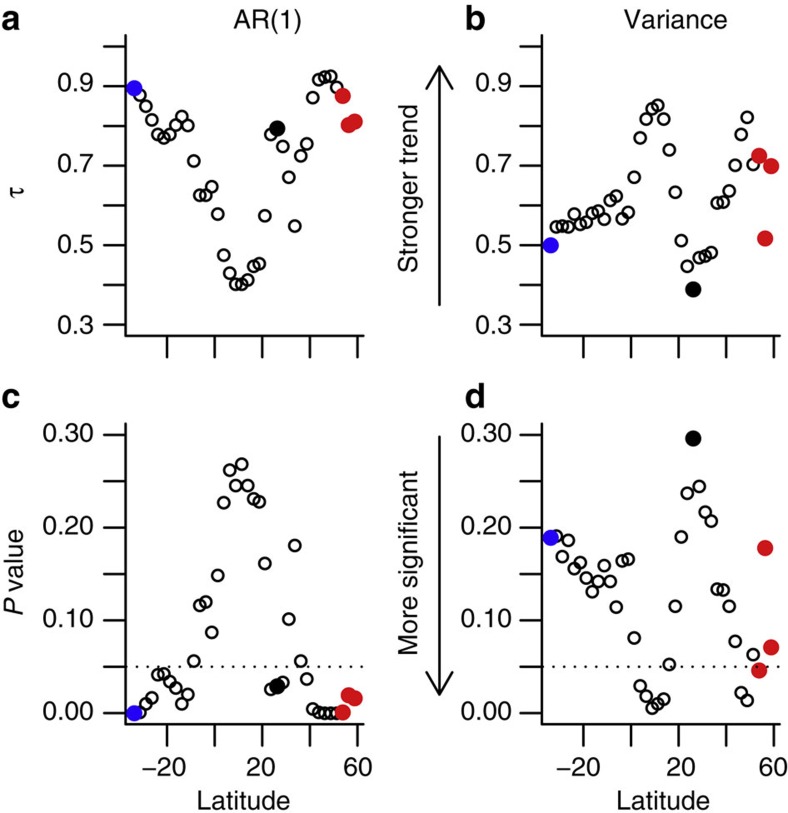
Tendency and significance of early warning indicators as a function of latitude. Kendall’s τ values are calculated to determine the tendency of estimated (**a**) AR(1) coefficient and (**b**) variance indicators (see main text and Methods). Significance of results, using bootstrapped null model ensembles to determine *P *values (see main text and Methods), are plotted for (**c**) AR(1) and (**d**) variance. Any *P* values below the dotted horizontal line are significant at the 95% level (*P*<0.05). Black filled-in points correspond to 26.25°N (where the time series analysed in Fig. 3 is obtained). Approximate locations of the OSNAP and SAMOC monitoring arrays are shown in red and blue, respectively.

We next looked for early warning signals of AMOC collapse at a wide range of latitudes in the model Atlantic basin (33.75°S–58.75°N), which show varying AMOC strength (Fig. 5a,b, where 26.25°N is indicated by the black line). At all latitudes we find increasing AR(1) and variance (Fig. 5c,e), with Kendall’s τ values of 0.40–0.92 and 0.39–0.85, respectively (Fig. 6a,b). The absolute values of the indicators vary with latitude, hence their increase can be seen more clearly when we look at their percentage change over time (Fig. 5d,f). There appears to be a degree of meridional coherence to the indicators based on the annual data; the most robust upward trends in AR(1) are found towards the southern boundary of the Atlantic and in the high northern latitudes (Fig. 6a), whereas the most robust upward trends in variance are found just north of the equator (Fig. 6b).

We test the significance of the early warning signals found by comparing them to a null model. A null model ensemble of 1,000 members was created using a bootstrapping method to create time series at each latitude that have similar characteristics—such as mean and variance—to the original time series (see Methods). Comparing the transient run to the null model, a hypothetical early warning system located at 26.25°N, near the latitude of the RAPID/MOCHA/WBTS array, provides an AR(1)-based early warning signal that is significant at the 97% level (*P*=0.029 at 26.25°N, Fig. 6c). Variance provides a less reliable early warning signal at this latitude (*P*=0.296 at 26.25°N, Fig. 6d). Looking across latitudes the most reliable early warning signals from AR(1) (Fig. 6c) are found in the mid-high northern latitudes (*P*<0.05 at 38.75–58.75°N with *P*<0.01 at 41.25–53.75°N) and at the southern boundary of the Atlantic (*P*<0.05 at 11.25–33.75°S with *P*<0.01 at 28.75–33.75°S), consistent with the locations where the largest values of τ are found. In the sub-polar North Atlantic, the AR(1) early warning signal is significant at the 99.9% level (*P*<0.001 at 51.25°N). The most reliable early warning signals from variance (Fig. 6d) are found in the equatorial North Atlantic (*P*<0.05 at 1.25–13.75°N), and in parts of the sub-polar gyre (*P*<0.05 at 46.25–48.75°N), again where the largest τ values were found. Overall, rising variance is a less reliable early warning indicator than increasing AR(1).

### Early warning signals of AMOC collapse using decadal means

**Figure 7:**
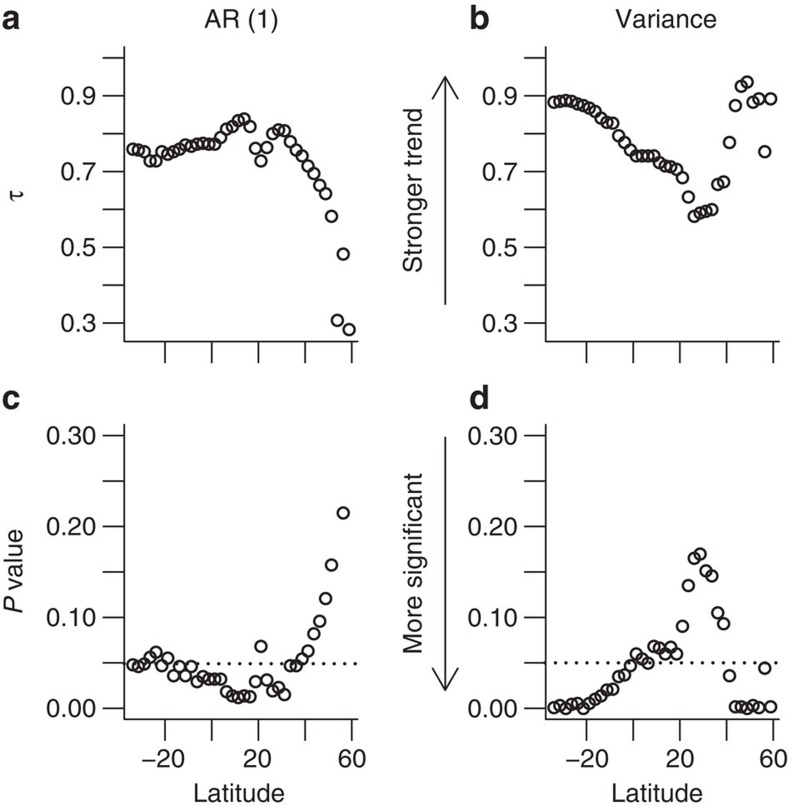
Tendency and significance of indicators for decadal resolution time series. As in Fig.6, Kendall’s τ values are calculated to determine the tendency of estimated (**a**) AR(1) coefficient and (**b**) variance indicators (see main text and Methods). Significance of results, using bootstrapped null model ensembles to determine *P* values (see main text and Methods), are plotted for (**c**) AR(1) and (**d**) variance. Any *P* values below the dotted horizontal line are significant at the 95% level (*P*<0.05).

When we test for early warning signals of AMOC collapse using the decadal means of the overturning circulation (Fig. 7), the statistics are expected to be poorer than from annual data due to using a time series with fewer points^27^. Nevertheless we still observe significant signals for both AR(1) and variance. Values of τ for variance are better than those of AR(1) with a range of 0.58–0.94 (Fig. 7b) compared with 0.28–0.84 (Fig. 7a). However, the latitudinal pattern of significance is different to that of the annual resolution indicators. Reasonably significant AR(1) signals are found in the tropics (generally *P*<0.05 for 33.75°S–36.25°N; Fig. 7c) but not in the North Atlantic (41.25°N and northward), whereas significant variance signals are found in the southern tropics and North Atlantic (*P*<0.05 for 33.75°S–21.25°N and 41.25–58.75°N and *P*<0.01 for 33.75–16.25°S, 43.75–53.75°N and 58.75°N; Fig. 7d).

### Comparison with signals from constant forcing simulations

As a further test of the significance of early warning indicators, the results from the transient run (at annual resolution) are contrasted with results from accompanying equilibrium simulations. The equilibrium simulations are initialized from specific points of the pre-collapse phase of the transient simulation, with the freshwater hosing maintained at a constant level^11^ until the system reaches equilibrium (black points in Fig. 3). In five of these equilibrium runs (at freshwater hosing values of 0.1, 0.12, 0.15, 0.2 and 0.22 Sv) the AMOC remains in the ‘on’ state for the full duration of the simulation (several thousand years in some cases). In another three equilibrium runs (freshwater hosing values of 0.25, 0.3 and 0.4 Sv) the AMOC eventually collapses (after hundreds of years). As none of these equilibrium runs is subject to a change in freshwater forcing, we do not expect them to show early warning signals due to there being no change in the stability of the underlying state.

**Figure 8:**
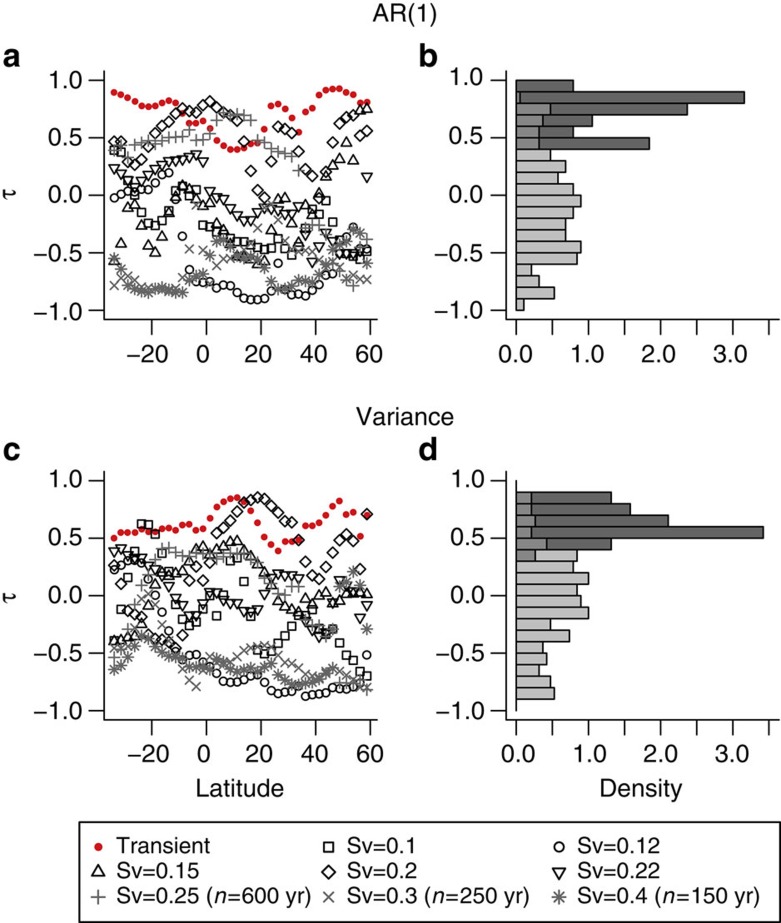
Comparing trends in early warning indicators from forced and equilibrium runs. Histograms comparing Kendall’s τ values from the annual resolution transient run with those from the equilibrium runs, combining the results across all latitudes (see main text and Methods) for (**a**,**b**) AR(1) coefficient estimation and (**c**,**d**) variance indicators. (**a**,**c**) Results from the transient run are shown in red, from equilibrium runs where time series equal to transient run length (*n*=800 years) could be obtained are shown in black and from equilibrium runs where a tipping point occurred preventing a long enough time series are shown in grey. Results are summarized in vertical histograms (**b**,**d**) respectively. In both cases, the lighter histogram is composed of τ values from the equilibrium runs and the darker τ values from the transient run with the intermediate shading implying the histograms overlap.

For each of these equilibrium runs, trends in the estimated AR(1) coefficient and variance are obtained at each latitude (see Methods), with the results shown in Fig. 8. As expected from the null models (Fig. 6a,b) the indicators from the equilibrium runs span a range of upward and downward trends (black and grey points in Fig. 8a,c). However, the trends in both AR(1) and variance are higher in the transient run than those from the equilibrium runs (red points in Fig. 8a,c) with a mean τ=0.71 for AR(1) in the transient run compared with τ=−0.17 across the equilibrium runs and τ=0.62 for variance in the transient run compared with τ=−0.16 for the equilibrium simulations. The distribution of τ values found in the transient runs for both AR(1) and variance (Fig. 8b,d) is significantly different from the distribution of τ values from the equilibrium runs when using a Mann–Whitney *U*-test (in both cases *P*<2.2 × 10^−16^). These results provide further evidence that the early warning signals in the transient run are real and have not occurred by chance.

### Length of time for indicators to become significant

**Figure 9:**
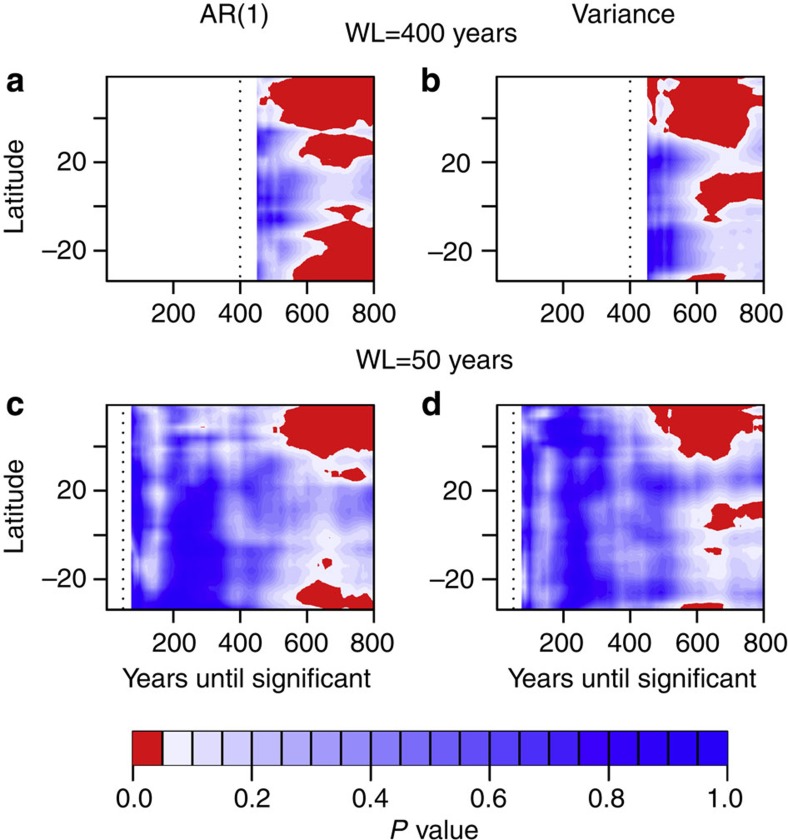
Time to significance of early warning indicators. Contour plots of significance as a function of latitude and the length of time series used are calculated as described in main text and Methods for window lengths of (**a**,**b**) 400 years and (**c**,**d**) 50 years (both shown by dotted line), using the annual resolution time series. Significance for each window length is tested after 50 and 25 years, respectively, to allow the indicators to be long enough to test significance on. Areas not shaded are where significance is not calculated due to either the length of time series at that point being less than the window length used (to the left of the dotted line), or the length of the indicator time series is less than 50 or 25 years depending on the window length used. Red shading suggests results are significant at 95% confidence (*P*<0.05) with blue shading not significant at this level.

To determine the length of time series needed to observe a significant signal and thus how far in advance the approach towards the tipping point might be predicted, we extend our use of the null models by testing against them on an increasing amount of data (Fig. 9; see Methods). We find that the signals begin to become significant (*P*<0.05, red in Fig. 9) after ~550 years of simulated data, 250 years before the tipping point occurs. Using two different window lengths (50 and 400 years), we find that for both AR(1) (Fig. 9a,c) and variance (Fig. 9b,d), the indicators become significant after using very similar lengths of data and in the same latitudinal regions (those seen in Fig. 6c,d).

## Discussion

Our results reveal generic early warning signals for a collapse of the AMOC in a fully coupled atmosphere-ocean general circulation model: the most realistic simulation of the climate system in which this type of signal has been tested. The hosing experiment carried out in FAMOUS involved a relatively slow, linear forcing. Nevertheless, comparison of the transient simulation with the equilibrium runs (Fig. 3) shows that the AMOC was forced fast enough to shift it away from equilibrium, such that it lagged the forcing (that is, collapse is delayed in the transient simulation). The theory of CSD is derived for systems close to equilibrium, yet it still seems to work in this case where the timescales of the forcing and the internal dynamics of the AMOC are comparable. In reality, anthropogenic forcing of the AMOC may be faster and more non-linear than simulated here. It is believed that recent freshwater forcing, over approximately the last 50 years, has increased by 0.026 Sv (ref. 28), which is comparable to the 0.05 Sv per century increase used to force FAMOUS here. However, anthropogenic forcing may increase faster in the future. It needs to be examined whether a more realistic forcing scenario can still produce early warning signals, or whether it eliminates them, as it does for another climate tipping element^29^. If anthropogenic forcing is faster than the intrinsic timescale of the ocean, then the early warning signals should not work as well as the system will not be near to equilibrium.

Our calculation of the length of time it takes for the early warning signals to become significant uses time series at annual resolution. In reality, palaeoclimate reconstruction of the AMOC would be required to gain enough data to begin to determine, if early warning signals are significant with enough time before collapse to be useful. Although existing palaeo reconstructions of the AMOC are at coarser temporal resolution than annual, our results also show that significant signals can be observed at a decadal resolution and thus potentially could also appear in these reconstructions^27^. Also the AMOC will not have been subjected to anthropogenic forcing for most of the paleo reconstruction era, which could be beneficial for observing a signal once the forcing begins. It has also been shown that the warm phase of the Atlantic multidecadel oscillation (AMO) coincides with a strengthening of AMOC and the cool phase, a weakening^30^ and multidecadal sea surface temperature (SST) variations are closely related to the AMOC in GCMs. The AMO has been reconstructed using SST records (including in-filling) since 1856 at a monthly resolution^31^ and using tree-ring palaeo data, it has been reconstructed at an annual resolution from 1567 (ref. 32). These reconstructions could act as a proxy for AMOC to test these early warning signals on, although caution should be used when comparing mean data at annual resolution from the model and annual SST reconstructions.

Dynamical systems theory suggests that CSD occurs due to the weakening of a restoring (negative) feedback as a tipping point is approached, causing an increase in the time taken for the system to recover from perturbations. In the case of the AMOC collapse in FAMOUS, the existence of CSD signals suggests that the gradual freshwater forcing is causing a negative feedback to weaken. An important stabilizing feedback on the AMOC involves changes in meridional heat transport^33^. A weakening of the AMOC leads to a reduction in northward ocean heat transport, causing a cooling of the high latitude North Atlantic and associated increase in density, which promotes a recovery of the circulation through increased deep water formation. However, as the freshwater forcing is applied in the transient experiments analysed here, the AMOC undergoes a gradual weakening (as can be seen in Fig. 3) before the collapse. In contrast to salinity anomalies, surface ocean temperature anomalies are strongly damped by atmosphere-ocean heat fluxes. This means that when the AMOC is weaker, with slower northward advection of surface water masses, the increased transit time allows increased damping of the temperature anomalies, weakening the negative feedback from the AMOC itself. This theoretical explanation for the CSD has perhaps the broadest applicability, but many previous studies have provided evidence for other, more detailed, restoring feedback mechanisms that are responsible for controlling the time scale of decadal-centennial variability in coupled AOGCMs. These include an ocean-only mode excited by atmospheric variability, in which heat and salinity transport both play a role and the overturning and gyre circulations interact^34^,^35^; a coupled ocean-atmosphere mode, in which AMOC variations trigger dynamical feedbacks in the atmosphere that act to oppose the AMOC anomaly^36^; feedbacks that involve shifts in the inter-tropical convergence zone, leading to salinity anomalies in the tropical Atlantic, which feed back onto the AMOC strength as they are advected northwards^37^; and feedbacks that involve links with the Arctic^38^,^39^. To properly understand the precise dynamical reason for CSD in FAMOUS, further work will be required to identify the dominant negative feedback that controls the time scale of AMOC variability in this model, and identify how it is influenced by freshwater forcing. As there is still debate about the key feedbacks that stabilize the AMOC in different AOGCMs, generalization of these results to other models and the real world remains an important challenge.

The early warning signals in the annual resolution data are most reliable in the high northern latitudes and towards the southern boundary of the Atlantic. Current monitoring of the AMOC occurs at 26.5°N, where in this model, early warning signals are somewhat less reliable. However, there are already plans to monitor the AMOC in the sub-polar North Atlantic with the OSNAP (Overturning in the Subpolar North Atlantic Program) monitoring array (~55–60°N, red in Fig. 6). There are also proposals for a SAMOC (South Atlantic Meridional Overturning Circulation) array located in southern boundary of the basin (~34.5°S, blue in Fig. 6). Our results based on annual resolution data suggest that these could be the best locations to try to diagnose trends in the dynamical stability of the AMOC. However, the latitudinal results are rather different with decadal averaged data (Fig. 7) and may well vary from model to model. Thus, at this stage we can only conclude that early warning signals are likely to be latitude dependent and therefore monitoring at more than one location may increase the likelihood of observing a robust early warning signal.

Latitudinal variation in the reliability of early warning indicators might potentially be understood in terms of the latitudinal characteristics of natural AMOC variability. There are several dynamical components to the AMOC^40^,^41^, whose relative contributions differ with latitude and have been diagnosed in a (100-year) control simulation of HadCM3 (ref. 42) (from which the FAMOUS model we use is derived). To leading order, the meridional velocity across a zonal section can be dynamically split into Ekman and geostrophic components. By definition, the Ekman component is surface-intensified and directly driven by the zonal mean wind stress. The geostrophic component can be further decomposed into baroclinic (vertical shear) and barotropic (depth-independent) contributions. The barotropic component arises due to an interaction between sloping topography and the component of the flow that is constant with depth. In an idealized basin with vertical sidewalls, the barotropic component of the overturning circulation would be zero. In reality, variations in ocean depth across the zonal section cause vertically constant flow to project onto the meridional overturning circulation. For example, a northward depth-independent flow in a shallow part of the section (for example, near the boundaries) and a southward depth-independent flow at longitudes where the ocean is deeper, when zonally and vertically integrated, would produce a net positive contribution to the meridional overturning transport. The remaining, baroclinic, component arises through thermal wind balance associated with zonal density gradients across the basin. There are several physical mechanisms that control basin-wide density gradients, including coastal wind-driven upwelling and downwelling^43^, local buoyancy forcing^44^ and changes in the formation rate and transport of remote water masses. The latter is of particular relevance to the present study, as a change in the density and transport of North Atlantic Deep Water (NADW) as it spreads southwards along the western part of the basin will be reflected in a change in the zonal density gradient and therefore the vertical shear and the baroclinic component of the AMOC^45^,^46^,^47^,^48^.

CSD occurs because a restoring feedback is weakening as a bifurcation-type tipping point is approached. This negative feedback involves the component of the AMOC that is thermohaline-driven and acting on multi-decadal to centennial time scales. It is likely that this will be reflected in the baroclinic (vertical shear) component associated with zonal density gradients, as this is the component in which changes in the density and extent of NADW are likely to be most strongly visible. Within the South Atlantic and at some latitudes in the northern North Atlantic, this baroclinic component dominates the AMOC^42^, and we speculate that this could be consistent with the enhanced significance of the early warning signal based on annual data from these locations. In the low-latitude North Atlantic, in contrast, there is a large influence from other components of AMOC variability, particularly that associated with strong, depth independent flow over sloping topography^42^, which may help to explain the reduced significance of the early warning signal there. However, a clear distinction between the dynamical drivers of the AMOC components remains elusive, and several studies suggest that the baroclinic and barotropic components can be closely linked. For example, the barotropic component can also be influenced by zonal density gradients, and it is clear that wind forcing plays a crucial role in sustaining the AMOC^49^.

In addition, it remains to be established whether the latitudinal variation in relative dominance of the dynamical components in HadCM3 (ref. 42) holds on longer (centennial) time scales, and in other models. Further work will be required to establish whether the latitudinal variation in early warning signal reliability exists in other models and to fully understand the dynamical reasons behind this.

While preparing this manuscript, another study was published that explores a different method for detecting early warning of AMOC collapse in the same model^50^. In that study, the indicators of CSD theory appear to fail to provide early warning of AMOC collapse. However, their analysis was unusual in looking for temporal spikes in the indicators and averaging the data over latitudes.

Generic early warning indicators can complement system-specific stability indicators, such as the sign of the freshwater transport by the AMOC at the southern boundary of the Atlantic^11^,^51^,^52^,^53^. This ‘*F*_ov_’ indicator may reveal whether the AMOC is in a bistable regime and thus give some indication of whether a sudden collapse is possible. The early warning signals discussed in the present study may complement such a bistability indicator by providing information about the approach of the system towards the tipping point. Historical reconstruction of variations in AMOC strength^54^,^55^, for example, based on fluctuations in North Atlantic SSTs^56^, will also be needed to establish natural variability and any trends up to the time of monitoring. Nevertheless, our results suggest that plans for new AMOC monitoring arrays could have a previously unrecognized value in helping establish whether the climate system is being pushed towards AMOC collapse.

## Methods

### Data

Data for the overturning are taken from the coupled climate model FAMOUS, which has been subjected to a hosing experiment, causing the AMOC to collapse^11^. The time series consist of the annual mean meridional overturning transport at ~1,000 m depth at each latitude. We determine that (at all latitudes) the AMOC begins to collapse after 800 years and so only use data up to this point in our analysis. For the majority of our analysis, we detrend using a Kernal smoother with a bandwidth of 100 years with the early warning signals tested on the residuals. To test the robustness of the indicators at 26.25°N, we vary the window length (see below) and detrending bandwidth used (Fig. 4e,f).

### Indicators and tendencies

AR(1) and variance are almost always tested on a moving window of length 400 years in our analysis (half the full-time series length). To calculate AR(1), we estimate *α* in the formula *x*_t+1_=*αx*_t_+*ση*, where *η* is Gaussian white noise of amplitude *σ*. The moving window shifts 1 year at a time, creating a time series for AR(1) and variance. Tendencies of these are measured by Kendall’s τ rank correlation coefficient, which for a time series of length *n* measures the proportion of concordant pairs to disconcordant pairs of data:


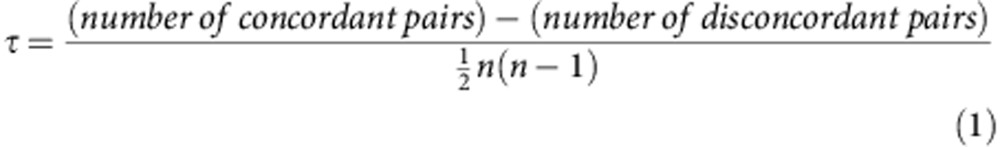


As one component is time that is always increasing, Kendall’s τ gives the trend of the indicator. A Kendall’s τ value of 1 indicates the AR(1) and variance are always increasing, −1, always decreasing and 0 indicates no overall trend.

### Null models and significance

We use null models to determine the significance of the early warning signals. A null model is created for each latitude, consisting of 1,000 members. Each ensemble member is the same length as the original time series pre-collapse (800 years) and is created by a bootstrapping method, sampling from the residuals of the time series (with replacement) once it has been detrended as described above. This ensures that each ensemble member has the same statistical properties as the original time series (that is, mean and variance), but the memory of the system is destroyed. Because of this, signals are not expected to be observed in the null model allowing us to explore the probability of the signal occurring by chance.

To determine a *P* value, we observe the proportion of the null model members that exhibit a higher Kendall’s τ value when AR(1) and variance are tested using the same window length (400 years). The *P* value is the probability of observing a signal as strong as that which we observe in the model by chance and any signal that has a *P* value <0.05 is considered significant.

### Time to significance

To determine the length of data needed to observe a significant signal, we use the null models as described above on a smaller section of the indicator’s time series. This section is then increased, 1 year at a time while testing the significance of the indicator each time. When using a window length of 400 years to obtain the indicators, we begin to test the time it takes for them to become significant after 50 years of indicator time series, and, for a window length of 50 years, we use 25 years. The time to significance is the number of years of indicator time series needed for the *P*-value to be <0.05, plus the window length, giving the total number of years of data needed.

### Equilibrium runs

For the equilibrium runs (see main text) that have no AMOC collapse, indicators are tested on the last 800 years of simulated data, again using a window length of 400 years and a detrending bandwidth of 100 years. This is to ensure that the influence of the previously increasing forcing is as small as possible. For equilibrium runs with AMOC collapse before the end of the run, the time series between the initialization from the transient simulation and the point of collapse are used (600, 250 and 150 years for the three runs in which this occurs) using window lengths equal to half the length of the time series but the detrending bandwidth maintained at 100 years.

## Author contributions

C.A.B. carried out the analysis with input from L.C.A. and T.M.L. T.M.L., L.C.A. and C.A.B. wrote the paper.

## Additional information

**How to cite this article**: Boulton, C. A. *et al.* Early warning signals of Atlantic Meridional Overturning Circulation collapse in a fully coupled climate model. *Nat. Commun.* 5:5752 doi: 10.1038/ncomms6752 (2014).
